# Synchrotron microbeam irradiation induces neutrophil infiltration, thrombocyte attachment and selective vascular damage *in vivo*

**DOI:** 10.1038/srep33601

**Published:** 2016-09-19

**Authors:** Daniel Brönnimann, Audrey Bouchet, Christoph Schneider, Marine Potez, Raphaël Serduc, Elke Bräuer-Krisch, Werner Graber, Stephan von Gunten, Jean Albert Laissue, Valentin Djonov

**Affiliations:** 1 Institute of Anatomy, University of Bern, Baltzerstrasse 2, 3012 Bern, Switzerland; 2 Institute of Pharmacology, University of Bern, Inselspital INO-F, 3010 Bern, Switzerland; 3 Université Grenoble Alpes, EA-Rayonnement Synchrotron et Recherche Medicale, ESRF, ID17 F-38043 Grenoble, France; 4 Biomedical Beamline, European Synchrotron Radiation Facility, BP220, F38043 Grenoble, France

## Abstract

Our goal was the visualizing the vascular damage and acute inflammatory response to micro- and minibeam irradiation *in vivo*. Microbeam (MRT) and minibeam radiation therapies (MBRT) are tumor treatment approaches of potential clinical relevance, both consisting of parallel X-ray beams and allowing the delivery of thousands of Grays within tumors. We compared the effects of microbeams (25–100 μm wide) and minibeams (200–800 μm wide) on vasculature, inflammation and surrounding tissue changes during zebrafish caudal fin regeneration *in vivo*. Microbeam irradiation triggered an acute inflammatory response restricted to the regenerating tissue. Six hours post irradiation (6 hpi), it was infiltrated by neutrophils and *fli1a*^+^ thrombocytes adhered to the cell wall locally in the beam path. The mature tissue was not affected by microbeam irradiation. In contrast, minibeam irradiation efficiently damaged the immature tissue at 6 hpi and damaged both the mature and immature tissue at 48 hpi. We demonstrate that vascular damage, inflammatory processes and cellular toxicity depend on the beam width and the stage of tissue maturation. Minibeam irradiation did not differentiate between mature and immature tissue. In contrast, all irradiation-induced effects of the microbeams were restricted to the rapidly growing immature tissue, indicating that microbeam irradiation could be a promising tumor treatment tool.

The major challenge of treating cancer is to achieve high treatment efficacy within the tumor while reducing collateral damage to the normal tissue. Synchrotron radiation therapy is a new tumor treatment strategy used at the preclinical stage^1^. Microbeam radiation therapy (MRT) is based on the fractionation into arrays of parallel beams, thus generating a spatially and periodically alternating dose distribution in the target. The normal tissue shows a remarkably high resistance when irradiated with microbeam peak doses up to 5000 Gray (Gy)^2^. For different synchrotron radiation therapies, a variety of beam widths have been proposed. Several variants have emerged within the past decades: microbeam radiation therapy^3^ and minibeam radiation therapy (MBRT), among others^4^. Both MRT and MBRT induced increases in the lifespan of tumor-bearing rodents and are proposed as potential tumor therapies^2^,^5^,^6^,^7^,^8^,^9^,^10^,^11^,^12^,^13^,^14^,^15^.

In MRT, 25–100 μm wide beams, which are typically separated by a few hundred micrometers (Fig. 1A), depose in-beam doses of several hundreds of Grays. For example, microbeam doses of 320 to 860 Gy, with a center-to-center distance of 200 to 400 μm, have been applied in several studies^10^,^16^, where a significant increase in the median survival time for different tumor bearing rodents was achieved. Different mechanisms have been proposed to explain the efficiency of MRT in tumor growth control, such as preferential damage on immature (tumoral) vessels^10^, intercellular communication between irradiated and less-irradiated adjacent cells^17^ or effects on the immune system^18^,^19^. The clinical use of MRT is currently limited by the fact that the necessary dose rates to apply the treatment without blurring the microbeams during the scan can only be generated by a synchrotron^6^,^20^,^21^,^22^. Therefore, MBRT was initially proposed as a trade-off to counteract some of the difficulties encountered with MRT. In MBRT, wider beams (500–700 μm) and lower doses than in MRT are commonly used^21^,^22^ (Fig. 1A). This procedure is believed to be tolerated by the normal tissue^6^,^21^,^22^. However, studies with a clear focus on the tissue sparing effect have not been published yet for MBRT.

In this study, we partially amputated the ventral half of the caudal fin 5–7 days before irradiation (IR) while the dorsal half was left untouched. Zebrafish possess a remarkable regenerative potential^23^ and are able to completely regrow the caudal fin^24^. The first week of regeneration is characterized by a rapidly growing, immature vascular network, which can be visualized *in vivo* by the presence of green fluorescent endothelial cells in the *Tg(fli1a:eGFP)*^*y1*^ zebrafish^25^. The fins were then irradiated in the dorsal-ventral axis with a single beam and a constant entrance dose of 5000 Gy (Fig. 1B). Single beams of six different widths used in MRT and MBRT were applied. The evolution of damage was assessed by *in vivo*-microscopy (images and movies), light and transmission electron microscopy. This allowed us to study irradiation-induced effects of micro- and minibeams on both mature and immature tissue in terms of blood perfusion, endothelial cell damage and immune response.

## Materials and Methods

All procedures related to animal care conformed to the guidelines of the Swiss government and were approved by the European Synchrotron Radiation Facility (ESRF) Internal Evaluation Committee for Animal Welfare and Rights.

### Study design

The dorsal caudal fin of 41 age-matched *Tg(fli1a:egfp)*^*y1*^ zebrafish (fluorescent endothelial cells) were partially amputated^25^. The fish were divided into seven conditions (control, 25, 50, 100, 200, 400 and 800 μm wide beams) and observed at three different time points (6, 48, 96 hpi). Additionally, 72 non-irradiated zebrafish were used to study *fli1a*^+^ blood cells.

### Caudal fin amputation

**Figure 1:**
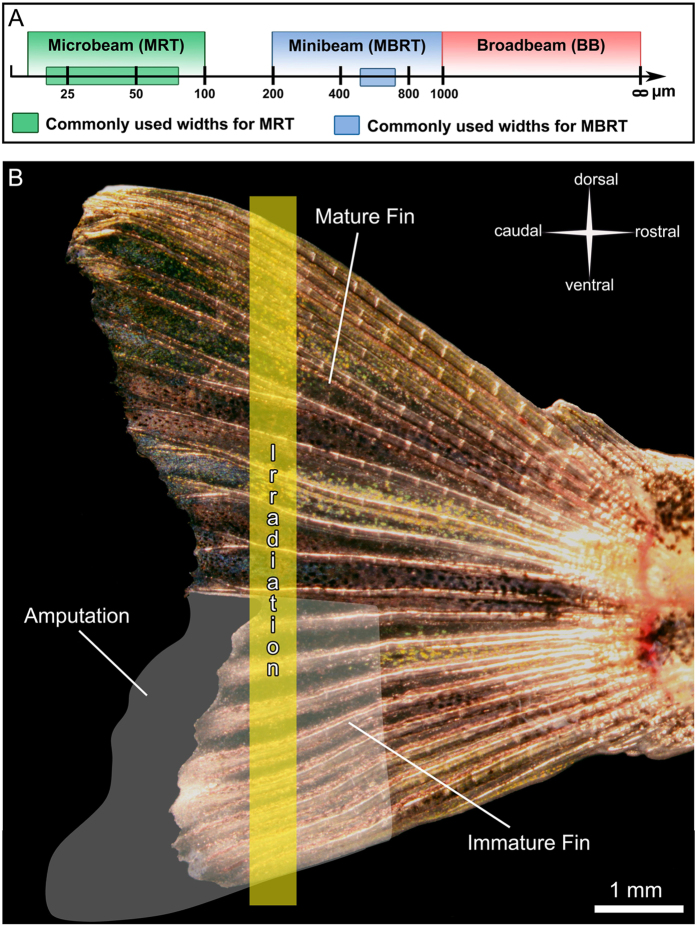
Irradiation of the zebrafish caudal fin. (**A**) Schematic representation of currently used beam types for preclinical radiotherapy studies. (**B**) Overview of a semi-amputated zebrafish caudal fin. Five to seven days post amputation, a single beam with a dose of 5000 Gy and a width of 25, 50, 100, 200, 400 or 800 μm was applied through both mature and immature parts of the fin.

The ventral half of the caudal fin of fish anesthetized with 0.04% MS-222 (500 μS, pH 7.4) was partially amputated using a scalpel (Fig. 1B) five days before the first set of irradiations.

### Synchrotron irradiation

Irradiations were performed at the ID17 biomedical beamline of the ESRF using X-rays. The caudal fin of zebrafish was irradiated 6 days after its semi-amputation. The fish were placed perpendicularly to the beam, positioned horizontally in a home-made Plexiglas support. A constant dose of 5000 Gy was applied with varying beam widths. The dose rate was ~16,000 Gy.s^−1^. We chose to apply 5000 Gy after preliminary experiments with 400, 1000 and 2500 Gy and based on the fact that zebrafish^26^ are up to 10 times more radio-resistant than humans^27^ or rats^28^.

### Dosimetry

The dose rate in MRT under broad beam conditions is determined by measurements using a calibrated pinpoint chamber (IC 31014 from PTW, Freiburg, Germany) following a protocol described in detail by Bräuer-Krisch *et al.* and Fournier *et al.*^25^. The delivered peak entrance dose is automatically calculated within the MRT GUI (Graphical User Interface), which applies Monte Carlo pre-calculated output factors for the different beam sizes scaled to the broad beam conditions at 2 cm depth for a 2 cm × 2 cm field size. Experimental dose verification with the help of Gafchromic film from ISP (Nuclear Associates) using a modified ZEISS microscope^25^ were equally performed.

### *In vivo*-microscopy

Zebrafish were anesthetized using 0.04% MS-222 in system water (500 μS, pH 7.4) for the irradiation procedure and *in vivo-*microscopy. We used a Leica M205FA stereomicroscope equipped with either one of two cameras. A Canon EOS 5D Mark II color camera with the EOS Utility software was used for brightfield investigations and movies. A Leica DFC365X grayscale camera with the Leica AF6000 software was used for z-stacks, movies and large mosaics.

### Flow cytometry, cell labeling and blood smears

For blood withdrawal, 72 non-irradiated zebrafish were anesthetized using 0.04% MS-222 in system water. Thereafter, a big portion of their tail was cut using a straight razorblade. The emerging blood was collected with a pipette and stored in 100 μl PBS in heparin-coated tubes (Microvette, Sarstedt, DE). For the frequency-assessment of the *fli1a*^+^ fraction, the blood was filtered with 40 μl cell strainers (Sigma-Aldrich, CH) and data directly acquired using FACSVerse (BD Biosciences). For the labeling of different surface cluster of differentiations (CD molecules), the cell isolates were fixed in 2% paraformaldehyde for 15 min, washed in PBS with 2% BSA (Sigma-Aldrich) and incubated with primary antibodies for 20 min at 4 °C. The secondary antibody was applied for 1 h at 4 °C. Stained cells were acquired using FACSVerse (BD Biosciences). The rabbit anti-zebrafish monoclonal antibodies against CD4 and CD8 were purchased from Anaspec, USA. The secondary donkey anti-rabbit AlexaFluor647-labeled antibody was purchased from Abcam, CH. FloJo Version 9.8.1 was used for data analysis. For blood smears, the blood was spread on a glass slide, air-dried for 3 min and then observed with a Leica M205FA stereomicroscope to identify *fli1a*^+^ cells. Thereafter, the cells were immediately stained with Giemsa-May Grünwald (Grogg Chemie, CH).

### Co-localization of *CD41* and *fli1a*

Co-localization of *CD41* and *fli1a* was determined in 5 dpf (days post fertilization) old fish with a Zeiss LSM880 confocal microscope. Ten pre-screened zebrafish were anesthetized using 0.04% tricaine and mounted on 35 mm glass-bottom petri dishes (MatTek, USA) using 0.8% agarose. 1000–1500 pictures of the posterior caudal vein (PCV) were acquired in and manually evaluated using Fiji-ImageJ v1.50 g.

### Blood perfusion at the tip of the caudal fin

The caudal fin consists of bony fin rays. Blood perfusion of each ray was scored as “yes” if the blood was able to pass the beam path in the artery, flow to the distal tip of the fin and back to the trunk. It was scored as “no” if the blood was either not flowing across the beam path (arterial damage) or if it was unable to flow back (venous damage). Blood perfusion was mainly assessed directly under the microscope, or using recorded movies.

### Light and transmission electron microscopy

Right after the *in vivo*-microscopy, the caudal fins with a big portion of the tail of anesthetized fish were cut off and immersed immediately in sodium cacodylate buffer (0.1 M, pH 7.4) with 2.5% glutaraldehyde (540 mOsm, pH 7.4), then washed three times for 8 minutes with 0.1 M sodium cacodylate buffer (pH 7.4) and post-fixed for 2 hours in the same buffer containing 1% osmium tetroxide (340 mOsm, pH 7.4). After another 3-step washing in the buffer the samples were dehydrated through a graded ethanol series and infiltrated with different aceton-epon mixtures. The caudal fins were divided into dorsal (mature) and ventral (immature) parts and embedded in epon 812 containing 1.5% accelerator. The samples polymerized over 3 days at 60 °C. Semi-thin sections for light microscopy (0.5–1 μm) and ultra-thin sections for TEM (70 nm) were obtained by an UCT Leica ultra-microtome with diamond knives from DIATOME SA. Semi-thin sections (0.5–1 μm) were observed under a light microscope (Zeiss Axio Imager M.2) with a mounted Olympus UC50 camera. Ultra-thin sections (70 nm) were observed with a transmission electron microscope (Philips TEM400).

### Statistical analyses

All statistical analyses were performed with GraphPad Prism v5.04. P-values of two-tailed paired t-tests were considered statistically significant if p < 0.05 (*p < 0.05, **p < 0.01, ***p < 0.001). Values are given as mean ± standard deviation.

## Results

### Microbeam irradiation leads to an early hemostatic and inflammatory response

**Figure 2:**
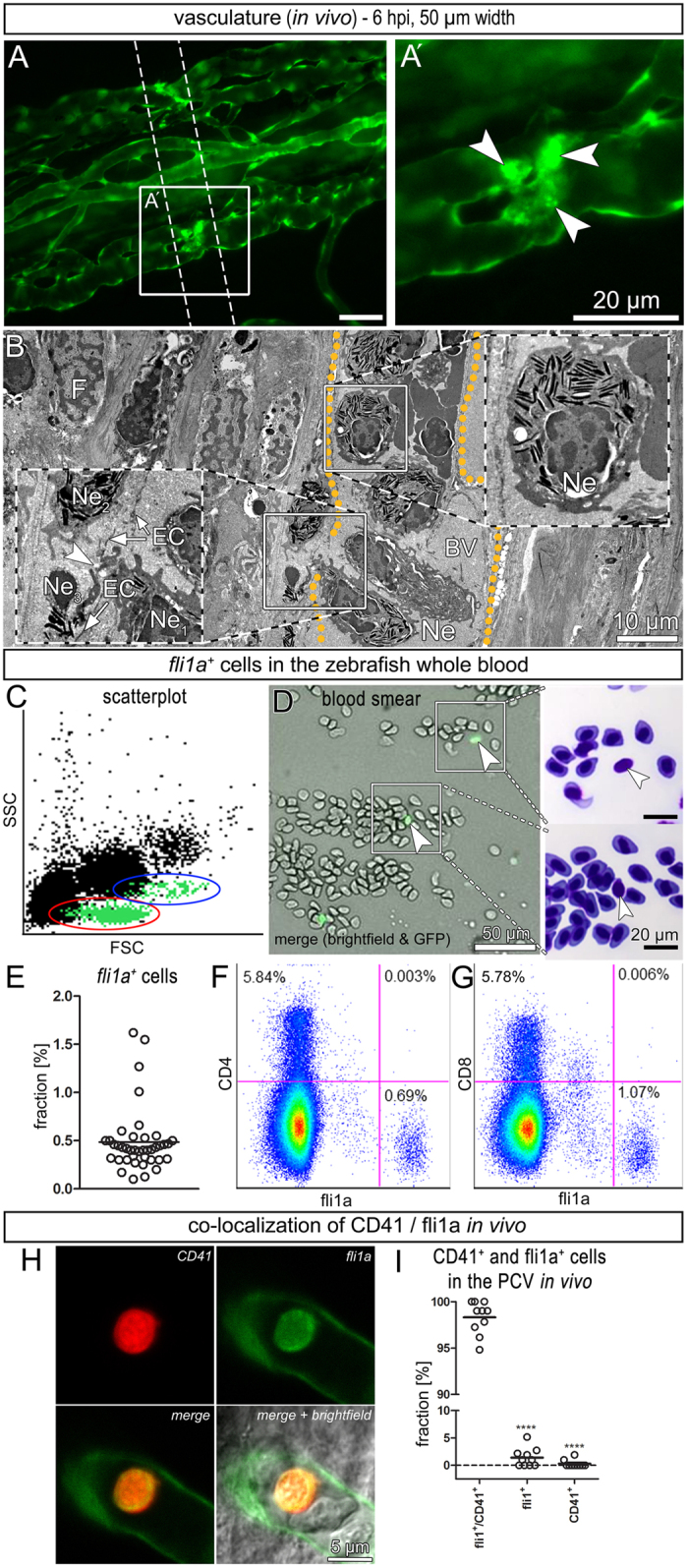
Early cellular response: local adhesion of neutrophils and thrombocytes (**A**,**A**’) Stereomicroscopic image showing that *fli1a*^+^ blood cells adhered to the venous wall inside the beam path (white arrowheads) *in vivo*. (**B**) Electron micrograph depicting an accumulation of neutrophil (Ne). (**C**) Scatter plot of peripheral blood with back-gated *fli1a*^+^ cells (green) clustering in two distinct populations. Red = lymphocytes and thrombocytes, blue = precursor cells. (**D**) Blood smear of untreated zebrafish whole blood. *Fli1a*^+^ cells were identified by fluorescence microscopy and relocated after staining with Giemsa-May Grünwald. (**E**) Fraction of *fli1a*^+^ cells in the whole blood (n = 40 [number of animals]). (**F**,**G**) Pseudo-color plots showing the absence of *fli1a*-expression in CD4^+^ and CD8^+^ leukocytes. (**H**) Example of a thrombocyte expressing CD41 and *fli1a* in 5 dpf old fish *in vivo*. (**I**) Fraction of CD41 and *fli1a* expressing blood cells in the posterior caudal vein *in vivo* (n = 10 [number of animals]).

We irradiated semi-amputated caudal fins of *Tg(fli1a:GFP)*^*y1*^ zebrafish with a single beam of 50 μm width and a dose of 5000 Gy (Fig. 1). Few hours after irradiation (IR), the beam path was visible as a fine line on the caudal fin. *Fli1a* is labeling endothelial cells and a subpopulation of blood cells. At 6 hpi (hours post irradiation), *fli1a*^+^ blood cells adhered to the endothelium in the beam path of the immature fin (Fig. 2A,A′) and continuously attached and detached (SI 1). These adherent cells coincided with neutrophils present in TEM micrographs (Fig. 2B). Neutrophils were identified by elongated protrusions and their characteristic electron dense, cigar-shaped granules, and were found in the blood as well as in the tissue (SI 2). We intraperitoneally injected LPS into adult zebrafish to increase the concentration of neutrophils in the blood. Blood smears have shown that these neutrophils did not express *fli1a* (SI 3). Previous research has delineated the different populations of zebrafish blood cells in scatter plots^29^. By the use of flow cytometry, we identified lymphocytes, precursor cells and thrombocytes that *fli1a*^+^ cells clustered in two different populations in the peripheral blood (Fig. 2C). Taken together, 0.49 ± 0.02% (n = 40 [number of animals]) of all blood cells were *fli1a*^+^ (Fig. 2E). In blood smears, *fli1a*^+^ blood cells appeared rounded, considerably smaller than the nucleated erythrocytes and with a barely visible amount of cytoplasm, thus suggesting a lymphocytic or thrombocytic origin (Fig. 2D). However, *fli1a*^+^ cells in the peripheral blood neither expressed CD4, nor CD8 (Fig. 2F,G). In a next step, we generated double-transgenic *Tg(CD41:GFP, fli1a:RedX)* fish and determined the co-localization of both fluorophores in the posterior caudal vein at 5 dpf *in vivo*. Among all fluorescent cells in the blood stream 98.31 ± 0.31% (500 quantified cells from 10 different animals) co-localized, 1.39 ± 0.29% were *fli1a*^+^*/CD41*^*−*^, and 0.29 ± 0.11% were CD41^+^/*fli1a*^*−*^ (Fig. 2I). These data strongly suggest that thrombocytes and neutrophils play early actions in opposing microbeam irradiation-induced damage.

### Immature tissue is damaged by both micro- and minibeams

**Figure 3:**
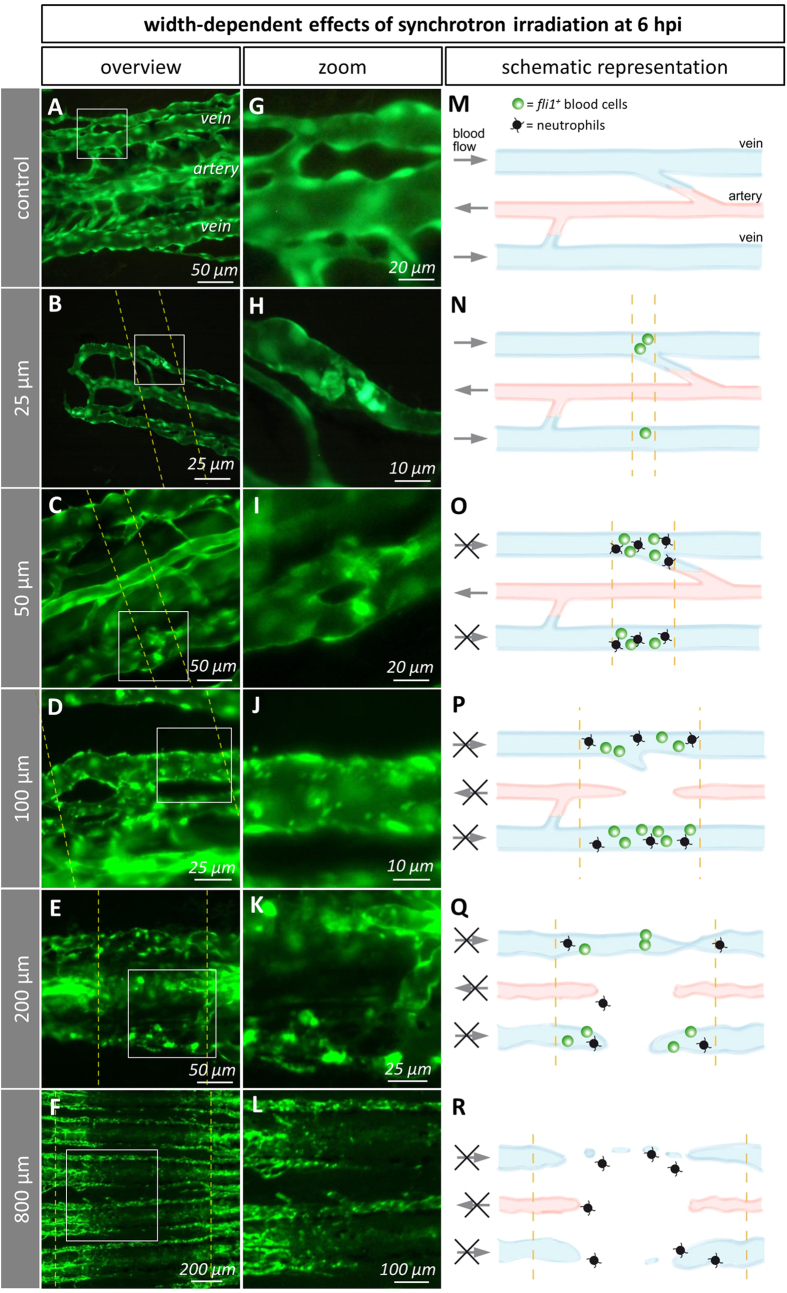
Width-dependent effects of IR on the immature caudal fin vasculature *in vivo* and as schematic representations. Dashed lines depict the beam path. Titles (left grey column) refer to the applied beam width. (**A**–**F**) Overviews. (**G**) Non-irradiated vein, 5 days post amputation. (**H**) 25 μm-wide beams induced adhesion of few *fli1a*^+^ thrombocytes in the beam path. (**I**) 50 μm-wide beams: adhesion of *fli1a*^+^ thrombocytes to the venous wall in the beam path. (**J**) Vein hit by a 100 μm-wide beam: About 1 μm sized spots of clumped, intensely fluorescent cells. (**K**) 200 μm-wide beams: Disrupted arteries and veins. (**L**) 800 μm-wide beams induced massive cell death inside the beam path. (**M**–**R**) Schematic representation of the beamlet width-dependent effects integrated from *in vivo*- (*fli1a*^+^ blood cells) and electron microscopy (neutrophils). Black arrows indicate the presence or absence (in case the arrow is crossed) of blood flow in arteries or veins. Accumulations of *fli1a*^+^ thrombocytes and neutrophils are indicated by green and black icons, respectively.

**Figure 4:**
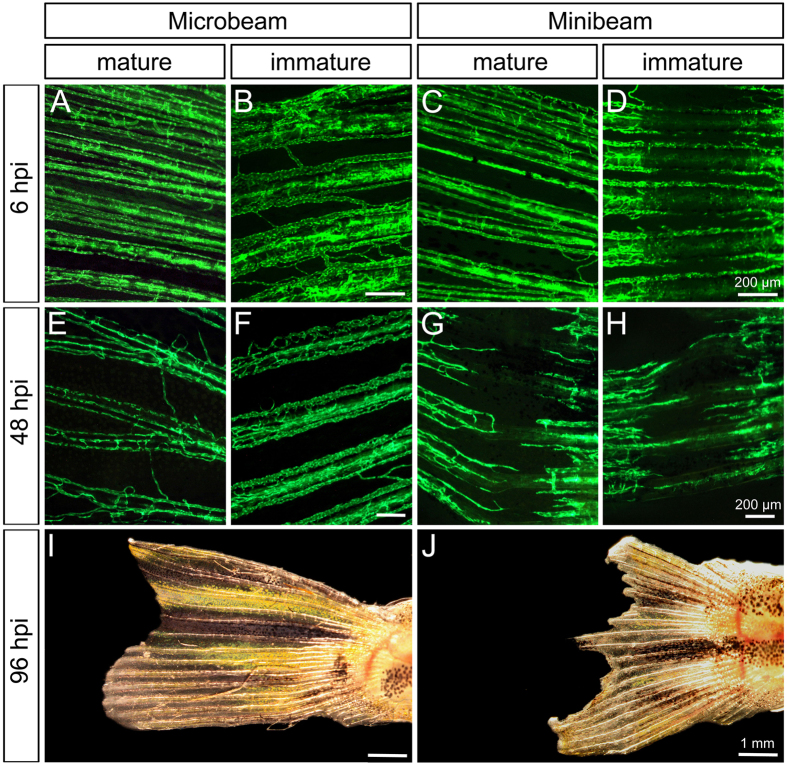
Effects of micro- (50 μm) and minibeam (800 μm) irradiation on the mature and immature vasculature. (**A**–**H**) Stereomicroscopic image of the vascular network *in vivo* at the site of irradiation. At 6 hpi (hours post irradiation), the mature vasculature was not affected by any type of irradiation. In contrast to microbeam-irradiated fish, massive vascular damage was visible in the mature fin of minibeam-irradiated fish at 48 hpi. (**I**,**J**) Brightfield images of whole caudal fins at 96 hpi.

We then asked whether the early cellular response in the immature fin is dependent on the beam width. Therefore, we irradiated semi-amputated caudal fins with micro- (25, 50, 100 μm) and minibeams (200, 400, 800 μm) with a constant dose of 5000 Gy (Fig. 1). Minibeam IR (200, 400, 800 μm) induced a loss of GFP-signal levels in the beam path, indicating local endothelial cell death at 6 hpi (hours post irradiation) in the immature vessels. In the 800 μm-group, the vascular network was discontinuous and displayed isolated endothelial cells (Figs 3F,L and 4D). The damaged tissue was infiltrated by neutrophils but we were unable to detect thrombocytes. In contrast to minibeams, microbeams did not cause the formation of a prominent gap in the vascular network (Fig. 4B,F). Beamlets of 200 μm width disrupted arteries and veins (Fig. 3E,K). In the 100 μm-group, most arteries were found to be discontinuous (SI 4). Noteworthy, the damage induced by exposure to microbeams narrower than 100 μm was restricted to veins. In the 25 and 50 μm-groups, accumulations of *fli1a*^+^ cells were observed in the vessel lumen at 6 hpi (Fig. 3B,C,H,I). Taken together, thrombocyte adhesion is widely restricted to vessels hit by microbeams whereas minibeams efficiently damage immature tissue.

### Mature tissue is damaged by minibeams but not by microbeams

At 6 hpi, the vasculature of the mature fin was affected neither by micro- nor by minibeams. At 48 hpi, irradiations with 400 and 800 μm wide beams induced a loss of GFP-signal levels in the beam path, as observed in the immature fin (Fig. 4G). Irradiations with 200 μm wide beams did not induce this effect, suggesting that the width necessary to cause a prominent gap in the vascular network lies between 200 and 400 μm (SI 8/9). At 96 hpi, the entire caudal fins irradiated with 800 μm were irreparably damaged and the necrotic tissue subsequently demarcated (Fig. 4J). Importantly, we did not observe any damage caused by microbeam IR in the mature vessels at any time point (Fig. 4A,E).

### Minibeams reduce blood perfusion in both mature and immature tissue

**Figure 5:**
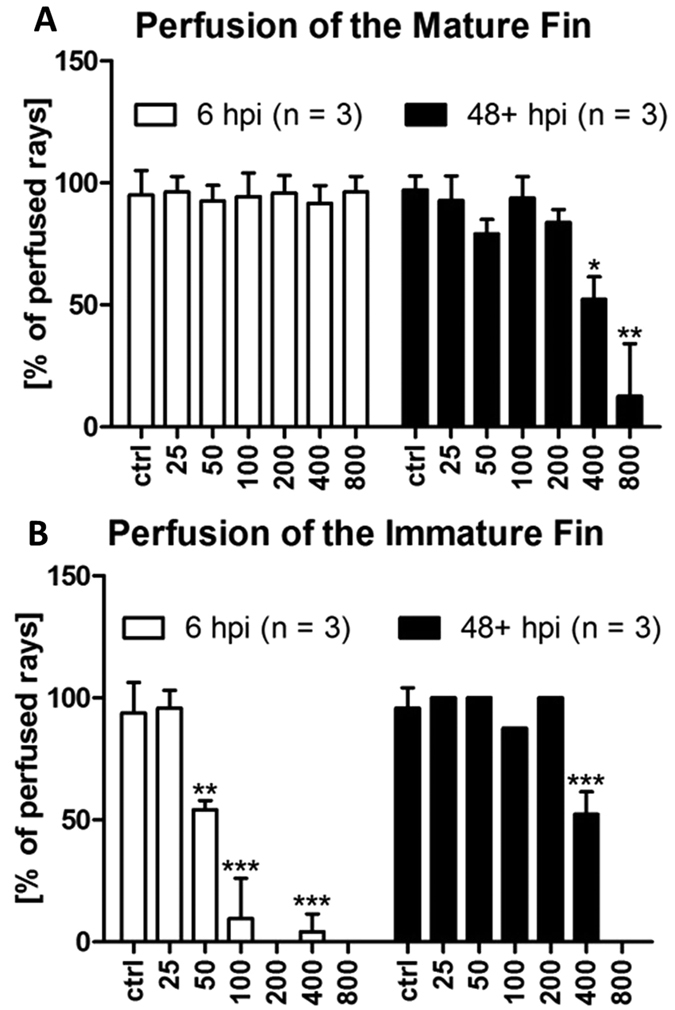
Blood perfusion of the caudal fin following irradiation. (**A**) Blood perfusion of the mature and immature fin at 6 and 48+ hpi (i.e. 48 and 96 hpi) as a functional parameter of blood vessels. At 6 hpi, perfusion of the mature fin was unimpaired. Only at later time points, minibeams (400 and 800 μm) decreased the number of perfused fin rays. In contrast, only 25 μm wide beams did not reduce blood perfusion in the immature fin. n = number of animals used for this analysis. Error bars represent standard deviations. P-values of two-tailed paired t-tests were considered statistically significant if p < 0.05 (*p < 0.05, **p < 0.01, ***p < 0.001).

To assess blood vessel functionality, we determined *in vivo* blood perfusion distally to the beam path. Few (<5%) fin rays were found to be unperfused in control animals. Blood perfusion was not observed distally to the site of minibeam IR (200, 400, 800 μm) in the immature tissue at 6 hpi (Fig. 5B). In contrast to microbeam IR, irradiations with 400 and 800 μm wide beamlets significantly reduced *(p* = *10*^−*4*^ (*400* *μm*)*, p* = 5 * 10^−*5*^ (*800* *μm*)) or even abolished blood perfusion at 48+ hpi in the immature (Fig. 5B), as well as in the mature fin (Fig. 5A).

### Microbeams selectively reduce blood perfusion in the immature tissue

Blood perfusion of fins irradiated with microbeams (50, 100 μm width) was significantly reduced in the immature tissue at 6 hpi (Fig. 5B). Beam widths of 50 μm reduced the blood perfusion distally to the beam path to 54.23 ± 3.06% (mean ± SD) and 100 μm widths to 13.47 ± 9.52% (Fig. 5B). An illustrative example of an IR of 100 μm width is shown in the supplementary information (SI 5). This effect can partially be explained by the accumulation of blood cells (mostly thrombocytes and neutrophils) in the vessel lumen (Fig. 2A,B) and, more importantly, by the interruption of vessels. Exposure to 25 μm wide microbeams did not affect blood perfusion. At 48 hpi and later, blood perfusion was found to be unaffected in all cases, which is most probably due to the zebrafish’s high regenerative capability (Fig. 5B). In the mature tissue, we did not observe any significant decrease of blood perfusion after irradiation with microbeams (Fig. 5A). Hence, microbeam IR with 50 and 100 μm wide beams selectively damaged the immature blood vessels and tissue (Fig. 5A,B).

### Damage caused to the immature connective tissue

**Figure 6:**
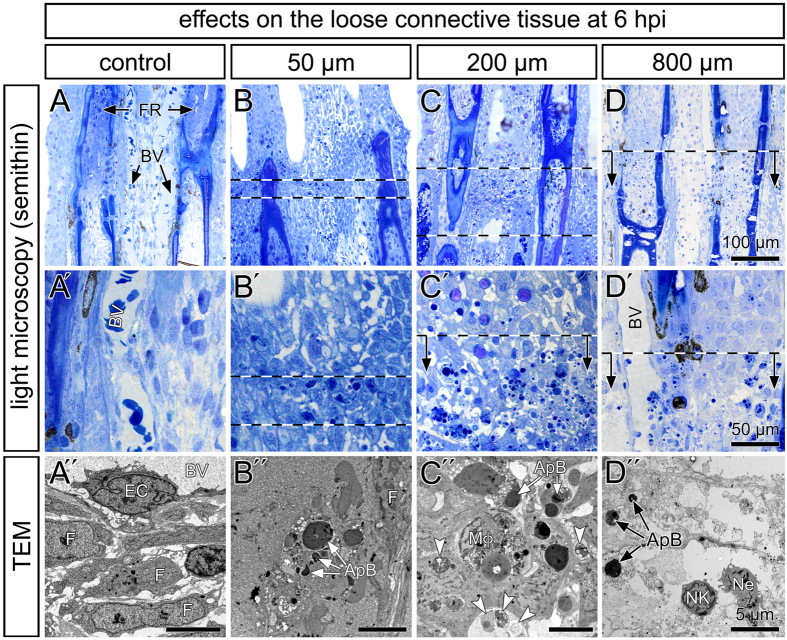
Structural alteration after micro- and minibeam irradiation. (**A–D**′) Semithin sections of the immature fins stained with toluidine blue. Shown are two neighboring bony fin rays (FR) and the loose connective tissue with blood vessels (BV). The site of irradiation is indicated by dashed lines and arrows. The cells in the beam path were characterized by solitary dark intracellular bodies and inclusions. Their number increased in the path of 200 μm wide beamlets. (**A**″–**D**″) Transmission electron microscopy demonstrated normal tissue appearance within the control fins with fibroblasts (**F**) of normal appearance, embedded in loose extracellular matrix and endothelial cells (**EC**). Electron-dense intracellular apoptotic bodies (ApB). Macrophages (Mφ) containing cellular debris and autophagic vacuoles (arrowheads). (**D**) The inter-ray tissue was disrupted and infiltrated by neutrophils (Ne).

Light and electron microscopy has been performed to visualize the damage caused to the rapidly growing tissue. Control animals display well-visible blood vessels lined by endothelial cells and surrounded by a loose connective tissue^30^ (Fig. 6A–A″). At 6 hpi, single cells in the path of 50 μm wide beamlets were characterized by nuclear fragmentations and dense apoptotic bodies in electron micrographs (Fig. 6B,B″). In the 200 μm-group, the number of cells containing apoptotic bodies and autophagic vacuoles was elevated (Fig. 6C,C″). These features are characteristic for cell apoptosis. In addition, tissue macrophages loaded with cell debris were observed (Fig. 6C″). In contrast, exposure to 800 μm wide beamlets induced tissue disruption with nuclear and cellular lysates, and the destruction of the plasma membrane leading to the disposal of cytoplasmic components and cellular organelles in the extracellular space. The acute cellular response is mainly characterized by neutrophils (Fig. 6D,D″).

## Discussion

We investigated high dose micro- and minibeam IR on mature and immature tissue with an emphasis on the vascular damage, cellular toxicity and acute inflammatory response. Microbeam (MRT) and minibeam radiation therapies (MBRT) for tumors are approaches of potential clinical relevance, both consisting of parallel X-ray beams that allow the delivery of thousands of Grays in their path.

The occurrence of vascular damage in the immature tissue depends on the beam width, which is summarized in SI 6. Beams wider than 100 μm disrupted arteries and veins (Fig. 3 and SI 4). At 48 hpi, these effects were no longer visible, which might be due to the regenerative capacity of the zebrafish. The effects of minibeams wider than 400 μm are not restricted to the immature part of the caudal fin and were particularly pronounced at 48 hpi. This suggests that minibeam IR potentially damages the normal tissue. In contrast, microbeam-irradiation induced vascular and tissue damage selectively in the immature vessels and tissue without affecting the mature part (Fig. 4).

We demonstrate that microbeams (50 and 100 μm width) significantly reduce blood perfusion and lead to an acute inflammatory response in the immature fin at 6 hpi restricted to the beam path. The immune system of zebrafish shows a high degree of overlap with the human system^29^ and is directly involved (namely macrophages and neutrophils) during caudal fin regeneration^31^. *Fli1a* is expressed not only in endothelial cells but also in certain blood cells of the zebrafish^25^,^32^,^33^,^34^. Neutrophil infiltration and the adhesion of *fli1a*^+^ blood cells coincided locally in the beam path of zebrafish irradiated with 50 μm-wide beams at 6 hpi. Accumulated *fli1a*^+^ blood cells appeared rounded, smaller than erythrocytes and express neither CD4 nor CD8. Co-localization of CD41/ *fli1a*^+^ blood cells was investigated in 5 dpf old zebrafish embryos, wherefore the direct translation of the quantified values to adult zebrafish has to be done with care. Nonetheless, CD41 and *fli1a*^+^ co-localized in 98.31 ± 0.31% (n = 10) of the circulating blood cells *in vivo*. A small proportion of CD41^+^ cells (10 out of 824 measured cells) did not express *fli1a* (Fig. 2I, movie in SI 7). It was previously shown that *fli1a* expression increases during thrombocyte maturation and that two populations of different fluorescent intensities exist^33^. Therefore, we hypothesize that the latter are either hematopoietic stem cells or, more likely, immature thrombocytes, which did not start to express *fli1a* yet. Taken together, these data strongly suggest an early involvement of thrombocytes and neutrophils in counteracting irradiation-induced endothelial cell damage.

In the light of our study, we add a hypothetical interpretation to the shape of the survival curves of intracerebral tumor bearing rats exposed to MRT^10^,^16^ and MBRT^6^,^22^,^35^,^36^. Minibeam-irradiated tumor-baring rats generally display a high initial survival rate followed by a rapid chute. This effect could be partially explained by a rapid tumor growth control, which is followed by substantial delayed toxicity to normal tissues. In contrast, survival curves in MRT-studies appear more widespread and decrease gradually.

To conclude, the present study grants novel insights into the effects depending on the beamlet width of collimated synchrotron-generated X-rays. This is the first comparison of microbeam and minibeam IR-induced vascular and tissue damage on different scale levels *in vivo*. We observed that high-dose beamlets wider than 400 μm may damage mature tissues, indicating a potential risk of such minibeams in tumor treatment. Exposure to 50 μm wide microbeams damages the loose connective tissue and blood vessels only in the immature fin, and leads to the recruitment of neutrophils and thrombocytes locally in the beam path.

## Additional Information

**How to cite this article**: Brönnimann, D. *et al.* Synchrotron microbeam irradiation induces neutrophil infiltration, thrombocyte attachment and selective vascular damage *in vivo*. *Sci. Rep.* **6**, 33601; doi: 10.1038/srep33601 (2016).

## Supplementary Material

Supplementary Information

Supplementary Video 1

Supplementary Video 2

Supplementary Video 3

Supplementary Video 4
