# Supplementary material for: Synchrotron microbeam irradiation induces neutrophil infiltration, thrombocyte attachment and selective vascular damage in vivo
Source: Sci Rep. 2016 Sep 19;6:33601. doi: 10.1038/srep33601 (PMC5027521; doi:10.1038/srep33601)
Supplement: Supplementary Information [file srep33601-s1.pdf]

SUPPLEMENTARY INFORMATION

SYNCHROTRON MICROBEAM IRRADIATION INDUCES NEUTROPHIL INFILTRATION, THROMBOCYTE ATTACHMENT AND SELECTIVE VASCULAR DAMAGE *IN VIVO*

Daniel Brönnimann[1a], *Audrey Bouchet*[1a#], *Christoph Schneider*[2], *Marine Potez*[1] , *Raphaël Serduc*[3], *Elke Bräuer-Krisch*[4], *Werner Graber*[1], *Stephan von Gunten*[2], *Jean Albert Laissue*[1] *and Valentin Djonov**[1]

[1] Institute of Anatomy, Group Topographic and Clinical Anatomy, University of Bern, Baltzerstrasse 2, 3012 Bern, Switzerland

[2] Institute of Pharmacology, University of Bern, Inselspital INO-F, 3010 Bern, Switzerland

[3] Université Grenoble Alpes, EA-Rayonnement Synchrotron et Recherche Medicale, ESRF, ID17 F-38043 Grenoble, France

[4] Biomedical Beamline, European Synchrotron Radiation Facility, BP220, F38043 Grenoble

[a] authors contributed equally to this work.

# current address: Rayonnement Synchrotron et Recherche Médicale, EA 7442, Université Grenoble Alpes, 71 Avenue des Martyrs, 38000 Grenoble, France.

*Corresponding author: Valentin Djonov, Baltzerstrasse 2, 3012 Bern, Tel. +41 31 631 84 32 (djonov@ana.unibe.ch)

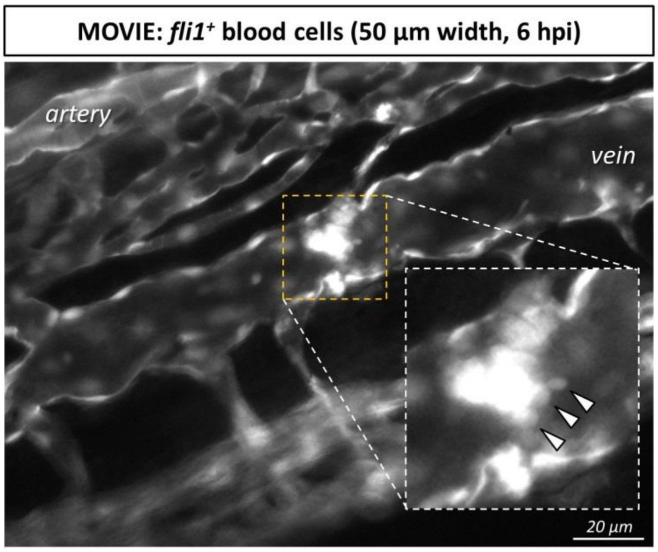

Supporting information 1. Attaching and detaching *fli1a*+ thrombocytes in the beam path previously irradiated with one 50 µm-wide beam. Real-time movie of a vein in the immature caudal fin at 6 hpi. *Fli1a*+ thrombocytes (white arrowheads) continuously attach and detach to the site of irradiation (orange encircled area).

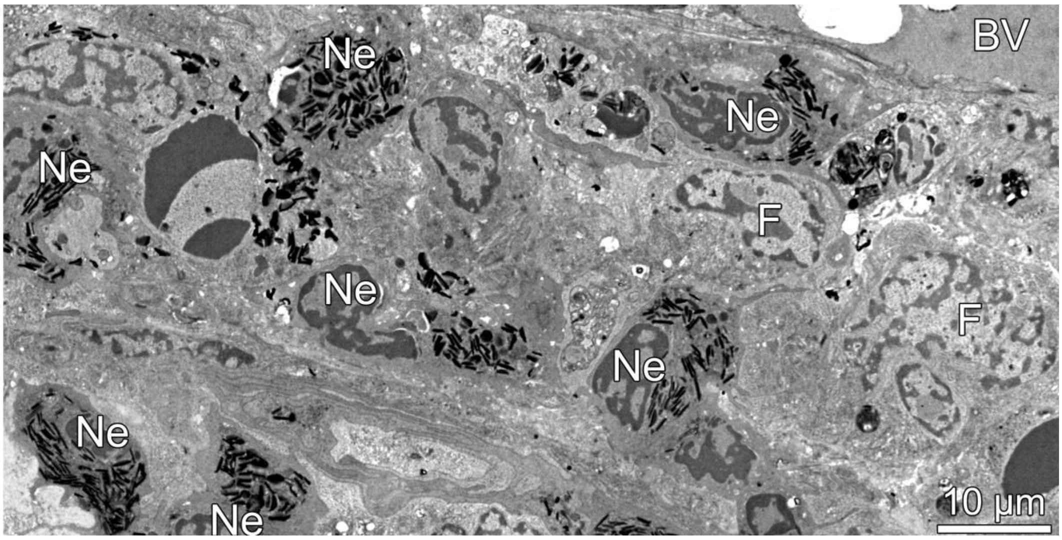

Supporting information 2. Extravasated and tissue-resident neutrophils (Ne) in the loose connective tissue. Neutrophils (Ne), fibroblast (F), blood vessel (BV).

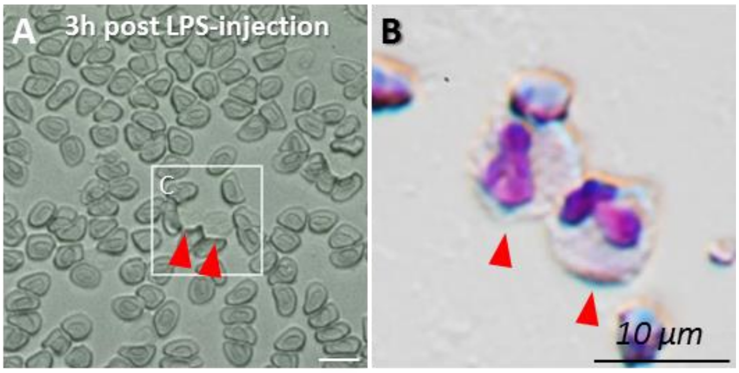

Supporting information 3. Neutrophils did not express *fli1a*. (A) Blood smear of zebrafish blood 3h post LPS-injection. (B) Neutrophils (red arrowheads) did not express *fli1*+.

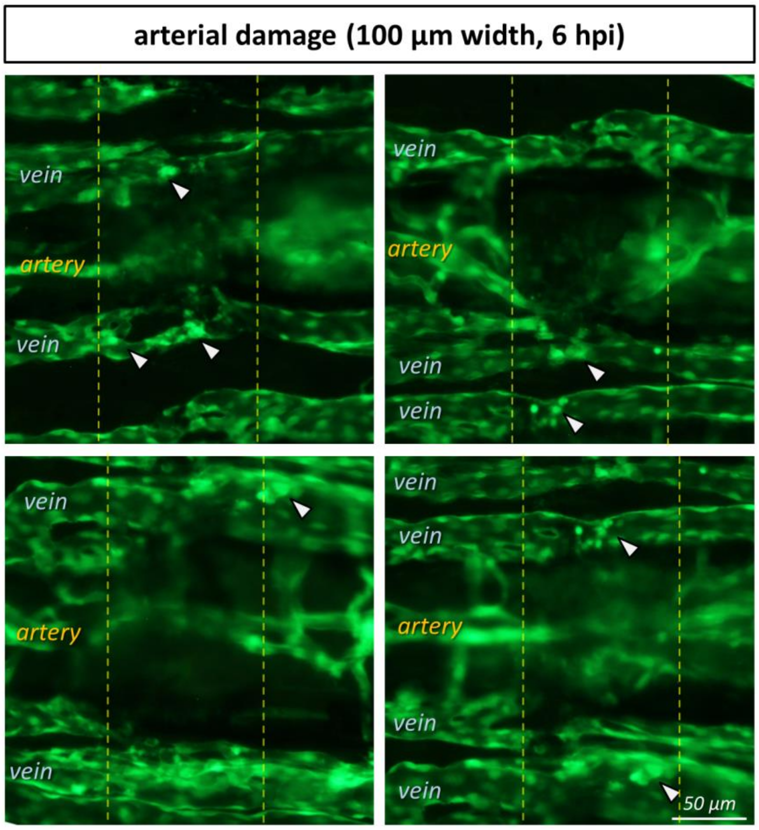

Supporting information 4. Microbeam IR with 100 µm-wide beams intersects arteries (6 hpi). Vasculature of fin rays irradiated with a 100 µm-wide beam. The yellow dashed line indicates the site of irradiation. Arteries are disrupted, whereas *fli1a*+ blood cells (white arrowheads) adhere to the vessel wall in veins sited in the beam path.

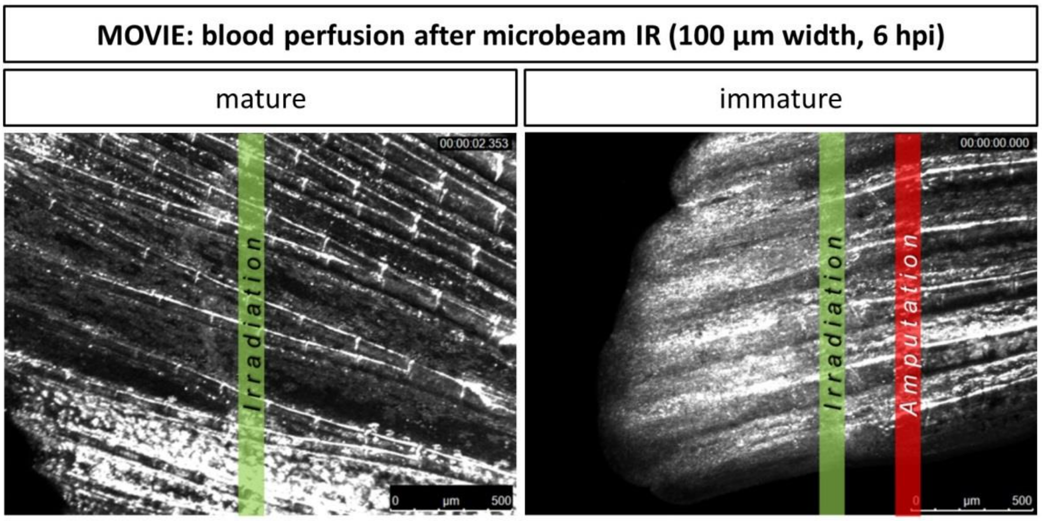

Supporting information 5. Blood perfusion of the mature and immature fin. In the mature fin, the blood flows past the irradiation site in both directions. In the immature fin, the blood vessels are discontinued, causing a defective blood perfusion distal to the site of irradiation.

6 hpi	mature fin						immature fin					
	25	50	100	200	400	800	25	50	100	200	400	800
Beam path visibility	minor	yes	yes	yes	yes	yes	yes	yes	yes	yes	yes	yes
Disturbed blood perfusion	no	no	no	no	no	no	no	yes	yes	yes	yes	yes
Adhesion of fli1+ cells	no	no	no	no	no	no	minor	yes	yes	N/A	N/A	N/A
Tissue damage	no	no	N/A	no	N/A	no	yes	yes	N/A	yes	N/A	yes
Fragmentation damage	no	no	no	no	no	no	minor	yes	yes	yes	yes	yes
Damage in veins	no	no	no	no	no	no	yes	yes	yes	yes	yes	yes
Damage in arteries	no	no	no	no	no	no	no	no	yes	yes	yes	yes

48+ hpi	mature fin						immature fin					
	25	50	100	200	400	800	25	50	100	200	400	800
Beam path visibility	no	minor	minor	minor	minor	yes	no	no	no	no	yes	yes
Disturbed blood perfusion	no	no	no	no	yes	yes	no	no	minor	minor	yes	yes
Damage in beam path	no	no	no	no	yes	yes	no	no	no	no	yes	yes
Damage distal to beam path	no	minor	minor	no	yes	yes	no	minor	minor	yes	yes	yes

Supporting information 6. Table summarizing the occurrence of the effects *in vivo*. (1) Beam path visibility: Whether or not the path was visible as a burnt-in line on the tissue. (2) Disturbed blood perfusion: Whether or not endothelial cell damage limited blood perfusion in the fin rays. (3) Adhesion of *fli1a*$^{+}$ cells: Whether or not *fli1a*$^{+}$ cells adhered to the endothelium within the beam path. (4) Tissue damage: Whether or not the surrounding tissue was visibly harmed; transmission electron microscopy findings. (5) Fragmentation damage: Whether or not the endothelial cells were characterized by dense fluorescent spots, possibly indicating membrane damage. (6) Damage in veins: Were venous vessels damaged? (7) Damage in arteries: Were arterial vessels damaged? (8) Damage in beam path: Whether or not the damage was restricted to the beam path. (9) Damage distal to the beam path: Whether or not the tissue distal to the beam path was visibly harmed (e.g. through prolonged lack of perfusion).

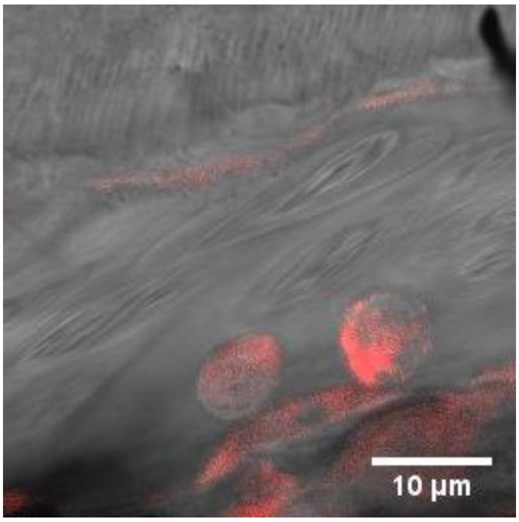

Supporting information 7. Movie of CD41^{+}/*fli1a*$^{-}$ blood cells rolling on endothelium in 5 dpf old fish.

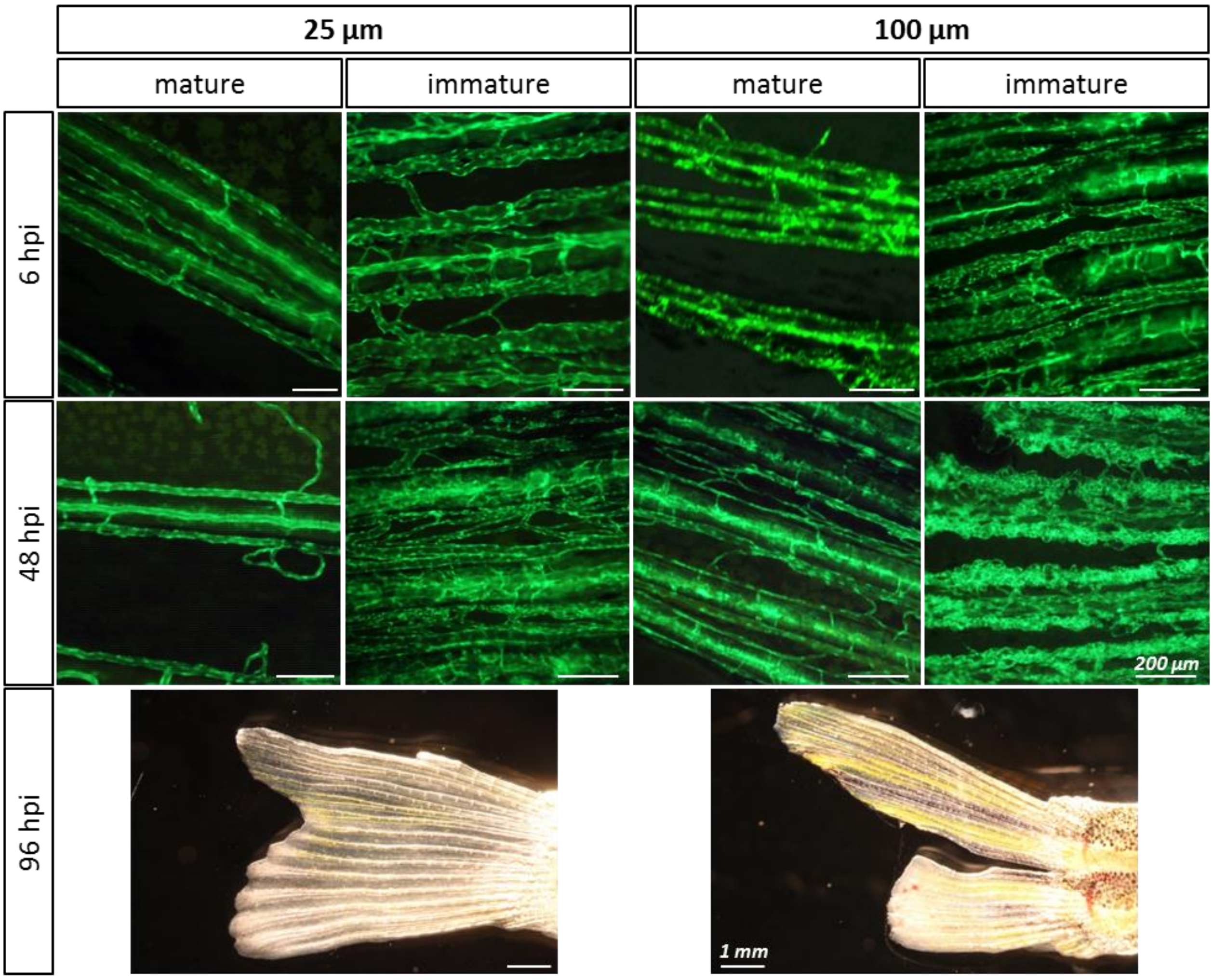

Supporting information 8. Effects of microbeam (25, 100 µm) irradiation on the mature and immature vasculature at 6, 48, and 96 hpi.

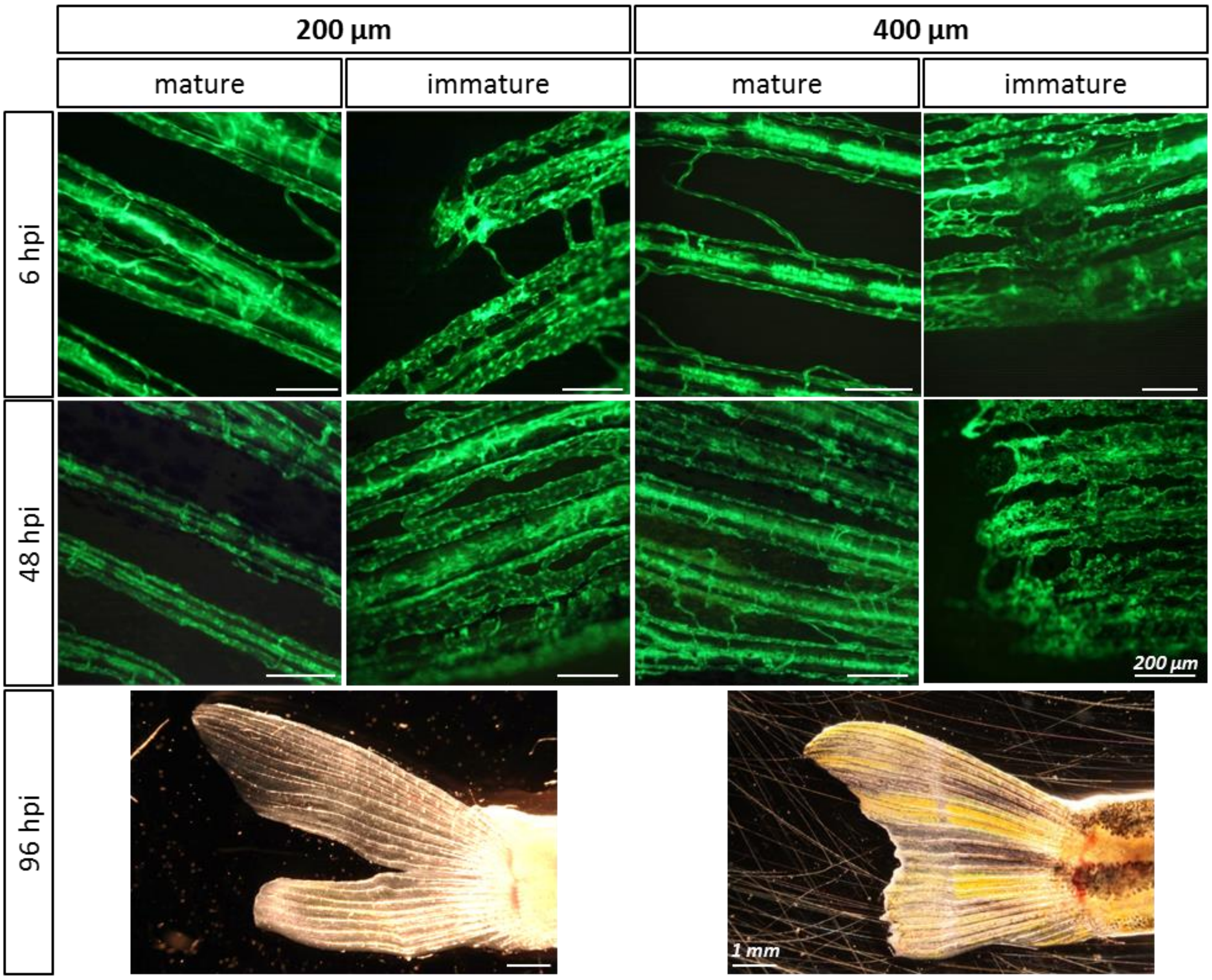

Supporting information 9. Effects of microbeam (200, 400 µm) irradiation on the mature and immature vasculature at 6, 48, and 96 hpi.
